# Effectiveness and safety of antihistamines up to fourfold or higher in treatment of chronic spontaneous urticaria

**DOI:** 10.1186/s13601-017-0141-3

**Published:** 2017-02-14

**Authors:** Mignon T. van den Elzen, Harmieke van Os-Medendorp, Imke van den Brink, Karin van den Hurk, Ouliana I. Kouznetsova, Alexander S. H. J. Lokin, Anna-Marijke Laheij-de Boer, Heike Röckmann, Carla A. F. M. Bruijnzeel-Koomen, André C. Knulst

**Affiliations:** Department of Dermatology and Allergology, University Medical Center Utrecht (G02.124), PO Box 85.500, 3508 GA Utrecht, The Netherlands

**Keywords:** Urticaria, Angioedema, Antihistamines, Refractory, Therapy

## Abstract

**Background:**

Treatment with second-generation antihistamines is recommended in patients with chronic spontaneous urticaria (CSU). Some patients remain unresponsive even after up-dosing up to fourfold. Many third line treatment options have limited availability and/or give rise to significant side effects. We investigated effectiveness and safety of antihistamine treatment with dosages up to fourfold and higher.

**Methods:**

This retrospective analysis of patients’ records was performed in adult CSU patients suffering wheals and/or angioedema (AE). Demographic, clinical, and therapeutic data was extracted from their medical records. We recorded the type, maximum prescribed dosage, effectiveness, and reported side effects of antihistamine treatment.

**Results:**

Of 200 screened patients, 178 were included. Treatment was commenced with a once daily dose of antihistamines. Persisting symptoms meant that up-dosing up to fourfold occurred in 138 (78%) of patients, yielding sufficient response in 41 (23%). Up-dosing antihistamines was necessary in 110 (80%) patient with weals alone or weals with angioedema and 28 (64%) with AE only (p = 0.039). Of the remaining 97 patients with insufficient response, 59 were treated with dosages higher than fourfold (median dosage 8, range 5–12). This was sufficient in 29 patients (49%). Side effects were reported in 36 patients (20%), whereof 30 (17%) experienced somnolence. Side effects after up-dosing higher than fourfold were reported in six out of 59 patients (10%).

**Conclusion:**

Up-dosing antihistamines higher than fourfold dosage seems a feasible therapeutic option with regards to effectiveness and safety. The need for third line therapies could be decreased by 49%, with a very limited increase of reported side effects.

## Background

 Chronic urticaria is either inducible (CINDU) or spontaneous (CSU) or both [1, 2]. Angioedema (AE) can occur concurrently with urticaria in up to 40% of cases, and may occur alone in up to 10–20% of cases [3]. Patients suffering CSU can have wheals only, AE only, or both [1].

The therapeutic approach of chronic urticaria aims at symptom relief. Licensed doses (1 tablet daily) of modern second-generation antihistamines (sgAH) are the first line treatment. An increase in the dose only up to fourfold is recommended as second line treatment [1, 4]. However, every third to fourth patient will remain symptomatic despite up-dosing up to fourfold [5], hence alternative treatments are needed for (partially) unresponsive patients [1]. Current third-line—in the US guideline fourth-line—treatment options consist of omalizumab, cyclosporine A (CsA) or leukotriene receptor antagonist montelukast [1, 4]. However, each of these options has limitations: omalizumab is expensive and not reimbursed worldwide. CsA has a high incidence of adverse effects. For leukotriene receptor antagonists, the level of evidence for efficacy is low [1].

In our tertiary center, refractory patients were treated with antihistamines at varying dosages (including dosages higher than fourfold), in order to avoid the use of CsA as omalizumab had not yet been approved for treatment of CSU. Despite a lack of controlled studies, experts have reported benefit of dosing antihistamines higher than fourfold in CSU patients [6]. The objective of this study was to investigate the frequency of ineffectiveness of treatment with antihistamines up to fourfold the standard dose in patients with CSU, and to determine the effectiveness and safety of antihistamine treatment above fourfold the standard dose,.

## Methods

### Study design and subjects

A retrospective analysis of patients’ records was performed in patients visiting our tertiary dermatology and allergology clinic for the evaluation of chronic urticaria and/or angioedema in 2012 (before registration of omalizumab), and for each patient all available data were collected up to 2014. Adult patients suffering CSU (wheals and/or AE for at least 6 weeks) were selected. All patients with other diagnoses including acute urticaria (duration of symptoms less than 6 weeks), CINDU including symptomatic dermographism, urticaria or angioedema caused by allergy or of other known causes, urticaria pigmentosa and urticaria vasculitis were excluded. Medical records were screened to verify inclusion and exclusion criteria. To be recognized as a representative sample, 159 patients were needed (based on a margin of error of 5%, a confidence interval of 95% and an eligible population of 268 patients) [7]. To have a representation of both AE patients and patients with wheals, all 100 available AE patients and 100 additional patients with wheals were screened for inclusion in the study. Patients with wheals were randomly selected based on their unique patient identification number in the electronic medical record system; dossiers of the patients with the lowest numbers were screened until 100 patients with wheals were included.

Data were collected as described below, and used in strictly anonymous form, according to the code of conduct for medical research approved by the hospital’s Medical Ethical Committee. Written informed consent for the publication of this report was not required from the patients, as approved by the Ethics Committee, protocol number 13-459.

### Treatment regimen

**Fig. 1:**
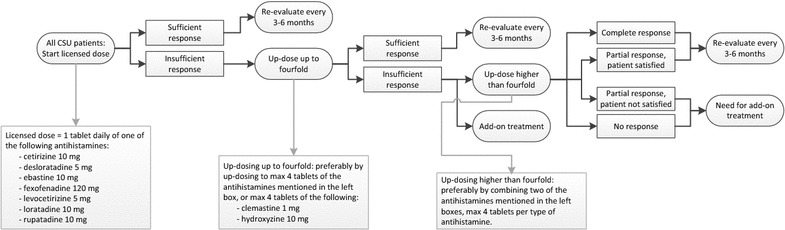
Local treatment protocol. When patients were already on antihistamine treatment prior to their first visit at the clinic, they could further follow the local protocol. Evaluation was planned every 3–6 months, or earlier if symptoms are intolerable

The local treatment protocol, as shown in Fig. 1, commenced with the approved dosage of antihistamine, and in case of persisting symptoms up-dosing occurred up to fourfold. Higher than fourfold dosages were only used in patients who remained symptomatic at fourfold antihistamine dosages. Treatment adjustments were performed individually by all prescribing physicians of the department. Patients often were already on antihistamine treatment prior to their first visit at the clinic. In this case, they did not have to start at the licensed dosage, but could further follow the local protocol. All treatment was open. At the start of the study, standard disease-specific questionnaires were not yet available and therefore not used.

### Data collection

After inclusion, data was collected from electronic patient records. Data regarding demographic and therapeutic characteristics until 2014 was extracted manually from the electronic medical records from each patient’s first visit to the clinic. Outcome variables were the type of antihistamines patients were treated with, the maximal prescribed dosage, treatment results, and reported side effects.

For each patient antihistamine use was recorded as daily treatment as well as rescue medication., The type, maximal prescribed dosage, treatment results including clinical symptoms of wheals, angioedema, and itch, and reported side effects were also recorded. Antihistamines were prescribed prior to or during consultations at this tertiary hospital. In the Netherlands, all types and dosages of antihistamines are reimbursed, hence prescribing is not affected by insurance. The doctor’s reported effect from each treatment option was allocated by the investigators into one of two categories: sufficient or insufficient. Disagreements were discussed and resolved. When the dose of antihistamines was raised and further information was missing, it was interpreted that lower doses did not reach sufficient response. The reported effect from dosages higher than fourfold was further subdivided into four different categories: (1) no effect (2) insufficient effect and patient not satisfied, (3) partial disease control, and patient satisfied, or (4) completely free of symptoms. If information was unclear category allocation was performed by two investigators. Up-dosing higher than fourfold was preferably performed by combining more than one type of antihistamine. In these cases the effect of one specific antihistamine was unclear and was not included for analysis. In case of side effects, the type of side effect as reported in the medical record, as well as the corresponding eliciting dosage of antihistamines, were recorded. Additional blood tests were not performed routinely.

### Analyses

Descriptive statistics were performed using IBM SPSS Statistics version 21. To explore differences in the proportion of patients with sufficient or insufficient effect from antihistamines in the three subgroups of patients (wheals only, AE only, and both wheals and AE), patients with unknown effect of treatment were excluded, and the Pearson Chi Square (Chi square) test was used. The Fisher-Freeman-Halton exact (Fischer’s exact) test was used in cases of low numbers.

## Results

### Population

Of the 200 screened patients, 178 patients (121 [68%] female; median age 48.2 years [range 20–87]) were diagnosed with CSU and were included in the study, including 10 patients who suffered both CSU and CINDU. Five of 200 were excluded due to angioedema with known causes (1 with HAE, 1 with specific allergy, and 3 with ACEi-AE) and 17 were excluded since they had only inducible symptoms (CINDU). Of the included 178, 43 patients (24%) had wheals only, 44 (25%) had AE only, and the remaining 91 (51%) suffered both symptoms. The median disease duration before the first consultation at our University referral center was 1 year (range 0–41.5 years). Ninety-four patients (53%) reported that they had previously visited another dermatologist or allergologist for evaluation of wheals and/or AE. All visits per patient were reviewed, and this comprised a median number of visits of 2 (range 1–57) and an additional median number of 2 consultations per telephone (range 0–24).

### Maximum doses and effectiveness of antihistamines

**Fig. 2:**
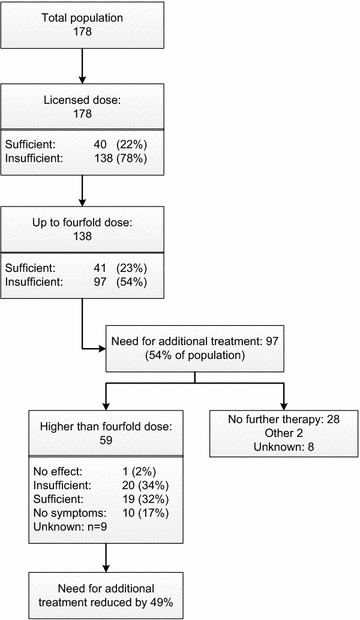
Antihistamine dosages and results. The following dosages were considered as standard dose: levocetirizine 5 mg, desloratadine 5 mg, fexofenadine 180 mg, clemastine 1 mg, hydroxyzine 25 mg, cetirizine 10 mg, loratadine 10 mg, acrivastine 8 mg three times daily

All 178 included patients were initially treated with the licensed, once daily, dosage of antihistamines (Fig. 2). Of them, 27 patients (15%) used antihistamines only on demand. In 138 patients (78%) the licensed dose was ineffective and in all these refractory patients the dose was raised up to fourfold. This remained ineffective in 97 (70%). Subsequently, 59 of these 97 patients were treated with higher doses of antihistamines by combining two types of second generation antihistamines with a maximum of eightfold the licensed dose. The median maximal combined dose of antihistamines in these 59 was eightfold (range 5–8), however in 8 individuals the dose was raised further (range 9–12). Ten of 59 patients (17%) subsequently became completely free of symptoms, and nineteen patients (32%) had sufficient results. Thus, in 49% of patients a higher than fourfold dose reduced or completely eliminated symptoms. The remaining 38 of the 97 refractory patients received no further treatment (n = 28), or further treatment was unknown (n = 8), or they received other types of therapy including ultraviolet (UV) treatment (n = 2), for which effectiveness results were not included in the current study (Fig. 2).

### Need for up-dosing in wheals versus AE without wheals

Up-dosing up to fourfold was necessary more frequently in patients with wheals (wheals only: 35 of 43 patients; 81%, wheals and AE: 75 of 91 patients; 82%) than in AE without wheals (28 of 44 patients; 64%; p = 0.039). However, when looking specifically at those with only one of the two symptoms, there was no statistically significant difference between wheals only and AE only (p = 0.053, Table 1a).

**Table 1:** Frequencies of up-dosing (a) and effectiveness of antihistamine dosages higher than fourfold (b)

*(a) Frequencies of up-dosing* ^a^				
Symptoms	Licensed dose n (%)	Up to fourfold n (%)	Higher than fourfold n (%)	Total n (%)
AE only	16 (36)	21 (48)	7 (16)	44 (100%)
Wheals only	8 (19)	18 (42)	17 (40)	43 (100%)
AE and wheals	16 (18)	40 (44)	35 (38)	91 (100%)

*(b) Effectiveness of antihistamine dosages higher than fourfold* ^b^				
Symptoms	Insufficient* n (%)	Sufficient n (%)	No symptoms n (%)	Total n (%)
AE only	2 (33)	2 (33)	2 (33)	6 (100%)
Wheals only	7 (58)	3 (25)	2 (17)	12 (100%)
AE and wheals	12 (38)	14 (44)	6 (19)	32 (100%)

* One patient suffering wheals only reported no effect of up-dosing to fivefold or higher, this case is included in the group of patients with insufficient effect. There was no statistically significant difference in treatment result between the three groups (Fischer’s exact p = 0.530) nor in those with wheals only (included for analysis: n = 17) and AE only (n = 7, Fischer’s exact p = 0.620)

^a^Percentages are shown per row to enable comparison between diagnoses groups. Patients are shown in their maximum dosage group, thus patients who received fivefold or higher have previously been treated with lower doses. Numbers therefore differ from Fig. 1. There was no statistically significant difference in frequency of up-dosing between the three groups (Chi square p = 0.053), and also not between those with wheals only (included for analysis: n = 35) and AE only (n = 28; Chi square p = 0.056). *n.a.* not applicable

^b^Percentages are shown per row to enable comparison between diagnoses groups. Effect of treatment was unknown in nine patients, the numbers of patients therefore differ from Table 1a

A trend was observed that up-dosing higher than fourfold was also necessary more often in patients with wheals (17; 40%) compared to AE only (7; 16%; p = 0.056, Table 1a).

Response to antihistamine dosages higher than fourfold dichotomized as sufficient (29 patients [58%]) versus insufficient (21 patients [42%]) did not differ between the three diagnosis groups (p = 0.530, Table 1b), or between patients with wheals only or AE only (p = 0.620).

### Types of antihistamines

The 178 patients received a total of 354 antihistamine prescriptions. As shown in Table 2, the most frequently prescribed antihistamines were levocetirizine (71% of patients), desloratadine (56%) and fexofenadine (23%). A total of 35 patients (20%) were treated with clemastine, and 26 patients (15%) were treated with hydroxyzine. Since 12 patients were treated with both hydroxyzine and clemastine at any time during their disease, a total of 49 patients (28%) received first-generation antihistamines (fgAH). All patients who were treated with fgAH were refractory to licensed doses: 13 (27%) received fgAH as part of up-dosing up to fourfold, and the remaining 36 (73%) received fgAH in addition to sgAH to reach total dosages of antihistamines higher than fourfold. Clemastine was up-dosed in 11 patients up to 3 mg per 24 h period, and hydroxyzine in nine patients up to 75 mg per 24 h.

**Table 2:** Frequency of use and frequency of satisfying result per antihistamine

Antihistamine	Frequency n (%)	Sufficient effect of licensed dose n (%)	Sufficient effect after up-dosing n (%)	Dose with sufficient effect median (range)
Levocetirizine 5 mg	126 (71)	15 (12)	26 (21)	2 (0–6)
Desloratadine 5 mg*	99 (56)	1 (1)	15 (15)	4 (1–6)
Fexofenadine 180 mg	41 (23)	5 (12)	2 (5)	1 (0–2)
Clemastine 1 mg	35 (20)	1 (3)	2 (6)	2 (1–2)
Hydroxyzine 25 mg	26 (15)	0 (0)	1 (4)	3 (n.a.)
Cetirizine 10 mg*	16 (9)	2 (13)	2 (13)	1.5 (1–4)
Loratadine 10 mg	9 (5)	0 (0)	1 (11)	2 (n.a.)
Acrivastine 3 × 8 mg	2 (1)	0 (0)	0 (0)	n.a.

Frequency data are presented as numbers and percentages of the total population (n = 178), and frequencies of sufficient response are presented as percentages of those treated with the specific antihistamine

* In 1 patient it was unknown which dose caused sufficient effect. *n.a.* not applicable. Please note that in most patients where up-dosing higher than fourfold occurred, this was done by combining more than one type of antihistamine. In these cases the effect of one specific antihistamine was unclear and was not included in this analysis

### Safety of antihistamines

**Fig. 3:**
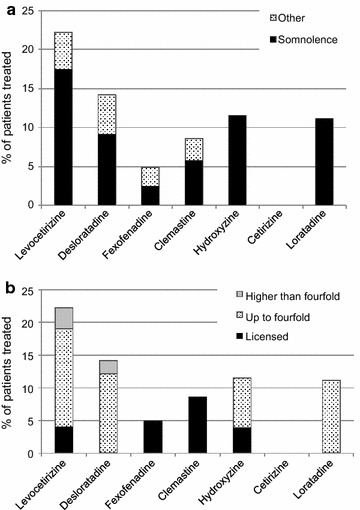
Frequency of side effects, by **a** type of side effect, and **b** maximum dose. The following dosages were considered as standard dose: levocetirizine 5 mg, desloratadine 5 mg, fexofenadine 180 mg, clemastine 1 mg, hydroxyzine 25 mg, cetirizine 10 mg, loratadine 10 mg, acrivastine 8 mg three times daily

Of the 178 patients 36 (20%) reported side effects upon treatment with antihistamines independent of the dosage. Fifteen of 36 patients reported side effects for two (n = 14) or three (n = 1) different antihistamines (Table 3). Somnolence (Fig. 3a) was reported in 30 of 36 patients (83%), including 5 patients (10%) treated with fgAH and 28 (16%) with sgAH. Six out of 36 (17%) reported side effects only during treatment with dosages higher than fourfold (Fig. 3b). They consisted of somnolence in five patients and were unclear in 1. Vomiting or diarrhea were not reported by any of the patients.

**Table 3:** Frequency of side effects per antihistamine

Antihistamine	Frequency n (%)	Somnolence n (%)	Other n (%)	Other side effects
Levocetirizine 5 mg	28 (22)	22 (17)	6 (5)	Weight gain (n = 2), palpitations, increase of symptoms, unclear (n = 2)
Desloratadine 5 mg	14 (14)	9 (9)	5 (5)	Palpitations, headache, increase of symptoms (n = 2), unclear (n = 2)
Fexofenadine 180 mg	2 (5)	1 (2)	1 (2)	Increase of symptoms
Clemastine 1 mg	3 (9)	2 (6)	1 (3)	Increased intra-ocular pressure
Hydroxyzine 25 mg	3 (12)	3 (12)	0 (0)	n.a.
Cetirizine 10 mg	0 (0)	0 (0)	0 (0)	n.a.
Loratadine 10 mg	1 (11)	1 (11)	0 (0)	n.a.
Acrivastine 3 × 8 mg	0 (0)	n.a	n.a	n.a.

Data are presented as numbers and percentages of patients treated with this antihistamine. Patients may have reported side effects upon treatment with more than one antihistamine, therefore the numbers do not match the total number of patients reporting at least one side effect. “Other” side effects occurred in one patient each, unless otherwise specified. Percentages are rounded and may therefore not match within one row. Please note that a low frequency of side effects may be due to a low frequency of use for the specific antihistamine, and to a lack of updating in the study population since only patient-reported side effects were shown

## Discussion

In CSU patients refractory to up to fourfold doses of antihistamines, higher than fourfold dosages reduced or completely eliminated symptoms in an additional 49%. Side effects were reported in 20% of patients and consisted mainly of somnolence. After up-dosing higher than fourfold only 6 out of 59 patients (10%) reported side effects.

In more than half of the total population response remained insufficient despite antihistamine treatment up to fourfold, consistent with previous studies [8–10]. Up-dosing higher than fourfold, with a median dose of eight tablets daily, was effective in half of patients including those with wheals only, with AE only, and with both symptoms. This is promising, since antihistamines have low costs as opposed to CsA or omalizumab, and they are available worldwide [1]. There is a lack of rationale for the dosage of fourfold being the maximum. In contrast, in both CSU and in cold urticaria, treatment with four tablets per day was shown to be more effective than three tablets per day, which in turn was more beneficial than two tablets or one tablet per day, indicating that higher doses could be more effective [9, 11]. Moreover, hydroxyzine is prescribed up to 200 mg/day, and because 30 mg of hydroxyzine equals about 10 mg cetirizine [11]. Two-hundred mg equals a dose of 60 mg cetirizine/day, considerably higher than fourfold. Since such high dosages of hydroxyzine are used in daily practice, it was likely that higher dosages of other antihistamines could also be effective. Additionally, hydroxyzine is a first generation antihistamine with considerably more side-effects than cetirizine. We conclude that many patients indeed had a favorable response to higher doses of antihistamines when doses up to fourfold were insufficient.

The effect of antihistamines, but only up to fourfold, has been studied previously, but very few head-to-head studies have been performed [9, 12]. Some studies have examined antihistamines up to fourfold [9, 13], or four tablets daily [8]. The latter may be somewhat confusing, for instance for fexofenadine where both 120 and 180 mg tablets are available. There are also studies available where only twofold dosages were the maximum [10]. Some studies showed preponderance of efficacy of higher dosages in the treatment of chronic spontaneous urticaria [8–10, 14], and cold and cholinergic urticaria [15–18]. In contrast, in some other studies comparable efficacy of standard and higher dosages was found [19–22].

The most frequently used antihistamine were sgAH. The use of fgAH is discouraged in the European guideline [1] since serious side-effects of these old sedating antihistamines have been reported, including lethal overdoses. Additionally, in the elderly they increase the risk of impaired cognition, inattention, disorganized speech, altered consciousness, and falls [1]. Yet, a substantial number of patients was treated with fgAH at some time during their disease: clemastine was prescribed to 20% and hydroxyzine to 15%. It was previously suggested that some physicians were not fully aware of the content of the most recent guidelines and therefore did not follow them [23]. However, the successful use of fgAH after failure of treatment with sgAH has been described [24]. Furthermore, the US guideline does support the use of fgAH in patients who do not achieve control of their condition with higher-dose second-generation antihistamines [4]. Our results support that the addition of not only sgAH but also of fgAH can lead to sufficient disease control when either licensed doses of sgAH, or dosages up to fourfold had failed.

Somnolence was reported by a minority of patients. It is well known that somnolence is one of the most reported unwanted effects of antihistamines. It occurs even when using sgAH [9] in up to 23% of patients [8], and it does not significantly increase when comparing with baseline somnolence [9], or when antihistamine doses are increased [8]. This was confirmed in our study for even higher dosages. Patients treated with fgAH did not report sedation more often than those treated with only sgAH. A possible explanation for this is that fgAH were mostly used in low dosages in addition to high dosages of sgAH, whereas often times relatively high doses of fgAH are used [24–26]. Very few of the side effects (10%) were reported only when antihistamine dosages were raised higher than fourfold. For desloratadine it was previously shown that dosages up to ninefold did not lead to clinically relevant adverse effects [27]. The low frequency of somnolence in the current study is likely to be an underestimation of unwanted effects due to missing information or recall bias, and since patients were not all actively asked about side effects, including but not limited to somnolence. It could also be caused by tolerance to somnolence which can develop within 4 days of subsequent use of H1 antihistamines [24, 28]. It was hypothesized that this is caused by adapted neuropharmacological effects [28]. On the other hand, it remains difficult to distinguish somnolence caused by treatment from somnolence caused by sleep disturbances due to the disease [8, 9]. Pruritus is most bothersome during the evening and at night when it makes falling asleep difficult and wakes patients later in the night. This causes chronic fatigue with a direct impact on QoL and physical and emotional well-being [5]. Still, although the influence of prolonged treatment on somnolence may be limited, and improvement of urticarial symptoms reduces somnolence [9], urticaria patients report sleep difficulties almost twice as often as control subjects [29], and our results support that somnolence occurred in a minority of urticaria patients [8].

A limitation of this study is the retrospective design. Therefore, precise documentation of results of treatment was missing in some patients. Also, there was a lack of objective measurements of effectiveness. With regard to side effects, we presented these as collected from the medical records. Somnolence was the most frequently named side effect. Liver and kidney function tests were not performed routinely. However, the extent of missing information was rather limited and different results are therefore not expected. Furthermore, the EAACI/GA2LEN/EDF/WAO urticaria guideline does not recommend to combine antihistamines [1], since the mechanism of action of sgAH is similar and mixing different antihistamines would therefore theoretically not have additional benefits [12]. In the current study we combined different antihistamines. This was performed in case dosages higher than fourfold were given, to limit side-effects related to a specific antihistamine. Lastly, CSU is a self-limiting disease. In the current study spontaneous remission may have occurred and this would then be misinterpreted as effectiveness of treatment.

In conclusion, we show that by up-dosing antihistamines higher than fourfold, half of patients reached sufficient treatment response while causing a limited increase in side effects. The need for other third line therapies could be decreased considerably. These findings need to be confirmed in a prospective controlled study. The results are of special interest in case of side effects or contraindications to currently proposed third line treatments, or when they are locally not (yet) available.

## Abbreviations

AE: angioedema
CINDU: chronic inducible urticaria
CsA: cyclosporine A
CSU: chronic spontaneous urticaria
fgAH: first generation antihistamines
IH-AAE: idiopathic histaminergic acquired angioedema
InH-AAE: idiopathic nonhistaminergic acquired angioedema
sgAH: second generation antihistamines
